# A Survey of Methods for Constructing Rooted Phylogenetic Networks

**DOI:** 10.1371/journal.pone.0165834

**Published:** 2016-11-02

**Authors:** Juan Wang

**Affiliations:** School of Computer Science, Inner Mongolia University, Hohhot, China; Principal Investigator, NORWAY

## Abstract

Rooted phylogenetic networks are primarily used to represent conflicting evolutionary information and describe the reticulate evolutionary events in phylogeny. So far a lot of methods have been presented for constructing rooted phylogenetic networks, of which the methods based on the decomposition property of networks and by means of the incompatible graph (such as the CASS, the LNETWORK and the BIMLR) are more efficient than other available methods. The paper will discuss and compare these methods by both the practical and artificial datasets, in the aspect of the running time of the methods and the effective of constructed phylogenetic networks. The results show that the LNETWORK can construct much simper networks than the others.

## Introduction

The evolutionary history of species is traditionally denoted as a rooted phylogenetic tree. Each tree represents certain evolutionary information of the species (denoted as cluster, will be discussed in the following) [1–4]. When rooted phylogenetic trees are constructed by different methods or from different datasets, all of the evolutionary information represented by these tree are often conflicting. The conflicting evolutionary information cannot be expressed as a phylogenetic tree. However, phylogenetic network can represent the conflicting evolutionary information, which is a generalization of phylogenetic tree. It can also describe the evolution involving significant amounts of reticulate events such as recombinations, hybridizations, and horizontal gene transfers [5–9].

Phylogenetic networks can be divided into unrooted [10–15] and rooted networks [16–26]. Unrooted phylogenetic networks are mainly used to visualize conflicting evolutionary information. Rooted phylogenetic networks not only can represent conflicting evolutionary information implied phylogenetic trees, but also can describe the reticulate evolutionary events that species occurred during evolution [27]. There is a large body of research on rooted phylogenetic networks. Devising appropriate algorithms for constructing rooted phylogenetic networks from rooted phylogenetic trees has become an important field of research in molecular evolution. Recently a lot of scholars have focused on the research of the field, and developed a number of methods.

Dendroscope [28] is a program for constructing rooted phylogenetic networks, which unites some methods such as the cluster network [29], the galled network [30] and the CASS [22]. Among all of the methods, the CASS can construct simpler networks than other methods, but it is extremely slow for large datasets. And the networks constructed by the CASS are highly dependent on the order of input data, i.e. the constructed phylogenetic networks are generally different for the same dataset when input orders are different. Then Wang *et al* improved the CASS algorithm, and designed two algorithms: the LNETWORK [31] and the BIMLR [32]. The LNETWORK and the BIMLR are faster than CASS and have less influence of input data order.

In the following, we refer to rooted phylogenetic networks as networks, unless otherwise stated.

## Preliminaries

Given a set of taxa X. A subset of X (except both ∅ and X) is called a cluster. For two clusters *C*_1_ and *C*_2_ on X, if they are disjoint or one is a subset of the other, i.e. *C*_1_ ∩ *C*_2_ = ∅ or *C*_1_ ⊆ *C*_2_ or *C*_2_ ⊆ *C*_1_, we say that *C*_1_ and *C*_2_ are compatible, otherwise they are incompatible. Given a set of clusters C on X. If any two clusters in C is compatible, C is called compatible, otherwise it is incompatible. The incompatibility graph *IG*(C) = (*V*, *E*) of C is defined as an undirected graph with node set *V* = *C* and edge set *E*, where two clusters are connected by an edge if and only if they are incompatible.

Let *S* be a subset of X. The restriction of C to *S*, denoted by ${C|}_{S}$, is defined as the result of removing all of the taxa in X\S (i.e. the taxa in X but not in *S*) from each cluster in C. The incompatibility degree of C, denoted by d(C), is defined as the number of edges in IG(C). Let C be a set of clusters on X and *x* a taxon in X. The incompatibility degree of *x*, denoted by *d*(*x*), is defined as the result of subtracting the incompatibility degree of ${C|}_{X\backslash \{x\}}$ from that of C, i.e. $d\left(x\right)=d\left(C\right)-d\left({C|}_{X\backslash \{x\}}\right)$. If the incompatibility degree of a taxon *x* is maximal among all of the taxa in X, i.e. d(x)=max{d(y)|y∈X}, we call that *x* is the incompatibility taxon w.r.t. C. The frequency of a taxon *x* w.r.t. C, denoted by *f*(*x*), is defined as the number of clusters which contain *x*, i.e. f(x)=|{C∈C|x∈C}|.

A rooted phylogenetic network *N* = (*V*, *E*) on X is a rooted directed acyclic graph (DAG for short), and its leaves are bijectively labelled as X. The indegree of a node *v* ∈ *V* is denoted as *σ*(*v*). A node *v* is a reticulate node if *σ*(*v*) ≥ 2; otherwise it is a tree node; particularly, a tree node is a root node if *σ*(*v*) = 0. An edge *e* = (*u*, *v*) is a tree edge if *v* is a tree node, otherwise it is a reticulate edge. The reticulation number in a network *N* = (*V*, *E*) is ∑_*σ*(*v*)>0_(*σ*(*v*) − 1) = |*E*| − |*V*| + 1.

Given a cluster *C* and a rooted phylogenetic tree *T*. If there is an edge *e* in *T* such that the set of taxa reachable from *e* equals *C*, we say that *T* represents *C*. Given a network *N*, when connecting one incoming edge and disconnecting all other incoming edges for each reticulate node, if there exists a tree edge *e* such that the set of taxa reachable from *e* equals *C*, we say that *N* represents *C* in the softwired sense. Alternatively, if there is a tree edge *e* in *N* such that the set of taxa reachable from *e* equals *C*, we say that *N* represents *C* in the hardwired sense.

Let *N* = (*V*, *E*) be a network representing the set of clusters C. A cluster C∈C is often represented by more than one tree edge in *N* and a tree edge *e* ∈ *E* often represents more than one cluster in C. If there exists a mapping *ϵ* from C to the set of tree edges of *N*, where *ϵ*(*C*) is a tree edge representing *C* for C∈C, such that for any two clusters $C_{1},C_{2}\in C$, *C*_1_ and *C*_2_ lie in the same connected component of the incompatibility graph *IG*(C) if and only if *ϵ*(*C*_1_) and *ϵ*(*C*_2_) are contained in the same biconnected component of *N*. Then we call that *N* is decomposable w.r.t. C.

When constructing rooted phylogenetic networks from rooted phylogenetic trees, the methods first compute the clusters represented by the input trees, and then construct a rooted phylogenetic network representing all clusters.

The rooted phylogenetic networks can describe evolutionary history in the presence of reticulate events, such as horizontal gene transfers, hybridizations and recombinations. These reticulate events are rare in reality [33]. Accordingly, it is expected that the constructed network has the minimal number of reticulate nodes. Let *N* be a network constructed for the input cluster set C. Assume that $C^{{}^{\prime }}$ is the set of clusters represented by *N*. In fact $C^{{}^{\prime }}$ contains more clusters than C, i.e. $C⊊C^{{}^{\prime }}$. Here we define the redundant clusters $C_{0}$ of *N* as the clusters which are in $C^{{}^{\prime }}$ but not in C, i.e. $C_{0}=C^{{}^{\prime }}\backslash C$. In phylogenetic analysis, the taxa in a cluster are putative monophyletic. Consequently, the ideal situation would be $C_{0}=\emptyset$, i.e. all clusters represented by the constructed network would be the clusters represented in the input trees, and no others. Therefore, by means of parsimony principle, the best constructed network is one that minimizes the number of redundant clusters, which is based on the prerequisite that it has minimal number of reticulate nodes.

The following section will introduce the main methods for constructing rooted phylogenetic networks from rooted phylogenetic trees.

## Methods

So far, the main methods for constructing rooted phylogenetic networks from rooted phylogenetic trees are the cluster network, the galled network, the CASS, the LNETWORK and the BIMLR. The following will give a brief introduction to each method.

The cluster network is a method for constructing rooted phylogenetic networks, which is based on the Hasse diagram. Given a set of clusters C. It first defines a partial order which is a binary relation ≼ on C: for u,v∈C, *u* ≼ *v* if and only if *u* ⊆ *v*. The (C,⪯) is called a partially ordered set. Then it draws a Hasse diagram *H* = (*V*, *E*) for (C,⪯), which is a DAG with node set V=C and the edge set *E*, where there is an edge *e* = (*u*, *v*) if and only if v⪵u and there exists no other node *w* in *V* such that v⪵w⪵u. Finally it labels the leaves of *H* by the taxa of X and assigns the root of *H*. The result DAG is the rooted phylogenetic network representing C.

The galled network is a method based on the seed-growing algorithm. It first finds a set of taxa S⊂X by seed-growing algorithm such that the set $C^{{}^{\prime }}={C|}_{X\backslash S}$ is compatible. Next it constructs a rooted tree *T* for $C^{{}^{\prime }}$. Finally it attaches the reticulate nodes to *T* under a certain amount of constraints, where the labels of nodes which are children of reticulate nodes are the taxa of *S*. The constructed network represents C.

The CASS, the LNETWORK and the BIMLR are the methods based on the decomposition property of networks. They first find all non-trivial biconnected components $C_{1},C_{2},\cdots ,C_{k}$ of IG(C); and then construct the subnetwork for $C_{i}\left(1\leq i\leq k\right)$; next integrate those subnetworks into a final network. The difference among them is the construction of the subnetworks.

We have known that a network *N* represents an incompatible set of clusters. After removing all reticulate nodes from *N*, *N* becomes a tree representing a compatible set of clusters. However, the construction of networks is the inverse process mentioned above. Given a set of clusters C on X. We can construct a tree for C if it is compatible, otherwise, we first remove some taxa from X, such that the result set $C^{{}^{\prime }}$ is compatible, then construct a tree for $C^{{}^{\prime }}$, finally append the removed taxa to the tree under certain conditions.

The CASS, the LNETWORK and the BIMLR construct subnetworks by the above description. Assume that the taxa set of $C_{i}$ is $X_{i}$ (1 ≤ *i* ≤ *k*). When constructing the subnetworks for $C_{i}$, they first remove a few taxa of $X_{i}$ from each cluster in $C_{i}$, such that the result set $C_{0}$ is compatible, then construct a rooted tree *T* for $C_{0}$, next attach some new reticulate nodes to *T*, finally add a new leaf below each reticulate node and label it as the removed taxon. From the process, we can see that the removed taxa in $X_{i}$ are very pivotal.

The difference among the CASS, the LNETWORK and the BIMLR is the removed taxa. The CASS tries to remove a few taxa, i.e. by means of trial and error, it randomly removes some taxa, if it can construct a network representing the set of clusters, then it stops; otherwise it continues to remove other taxa. The LNETWORK removes the taxa computed by seed-growing algorithm. The BIMLR removes the incompatibility taxa with the maximal frequency. The CASS method aims at minimizing the number of reticulate nodes, while the LNETWORK and the BIMLR not only minimize the number of redundant of clusters but also let the reticulate nodes as few as possible.

When constructing networks, the LNETWORK and the BIMLR find all networks representing the cluster set, mainly to reduce the number of redundant clusters in the resulting network and lessen the influence of the input data order on the resulting network.

## Results

In order to survey the performance of those methods, we do the experiments using both the practical and artificial data. The paper [31] has compared the LNETWORK, the CASS, the cluster network and the galled network; and the results show that the LNETWORK and the CASS can construct much simpler networks than the others. Here we just compare the LNETWORK, the CASS and the BIMLR using both the practical and artificial data (https://sites.google.com/site/cassalgorithm/data-sets).

All experiments were performed on a computer with an Intel Xeon E5504 2.0GHz CPU, 8 GB RAM and 147GB HDD. The operating system was Debian 4.1 32bit with Java 1.6 installed.

The experiments are used to compare two main aspects of these methods, on the one hand, the influence of input data order (Table 1 shows the results), on the other hand, the complexities of the constructed network, i.e. the reticulation number and the number of redundant clusters (Tables 2 and 3 show the results).

**Table 1:** Results of LNETWORK compared with BIMLR in terms of influence of input data order.

Data		LNETWORK				BIMLR			
|C|	|X|	*n*	mean	min	max	*n*	mean	min	max
35	22	1	0	0	0	1	0	0	0
25	15	1	0	0	0	1	0	0	0
22	13	2	1	1	1	2	1.5	1.5	1.5
27	15	2	1	1	1	2	1	1	1
25	13	3	1.2	0.5	1.5	1	0	0	0
22	11	1	0	0	0	1	0	0	0
17	10	3	1.3	1	1.5	3	1.3	1	1.5
13	8	2	1	1	1	1	0	0	0
23	11	2	1	1	1	2	1	1	1
18	10	3	2.5	1.5	3.5	3	1.5	0.5	2.5
21	11	1	0	0	0	2	0.5	0.5	0.5
12	10	1	0	0	0	1	0	0	0
21	10	2	1.5	1.5	1.5	2	0.5	0.5	0.5
13	7	2	1	1	1	1	0	0	0
22	10	1	0	0	0	2	0.5	0.5	0.5
21.3	11.7	1.8	1.2	1.1	1.4	1.6	0.6	0.4	0.7

Note: *n* represents the number of constructed networks and mean, min, max represent, respectively, the mean, the minimum, the maximum of tripartition distances of those networks.

**Table 2:** Results of BIMLR, LNETWORK and CASS for the artificial datasets.

Data		BIMLR			LNETWORK			CASS		
|C|	|X|	*t*	*r*	*c*	*t*	*r*	*c*	*t*	*r*	*c*
14	4	1 s	3	0	1 s	3	0	1 s	3	0
30	5	3 s	4	0	2 s	4	0	2 s	4	0
62	6	11 s	5	0	18 s	5	0	11 s	5	0
42	10	2 s	4	8	1 s	4	14	10 s	4	34
39	11	2 s	5	8	38 s	6	18	21 s	5	7
61	11	3 s	5	11	15 s	6	43	1 m 26 s	5	48
75	30	1 s	2	19	1 s	2	19	4 s	2	19
180	51	4 s	2	0	4 s	2	0	40 s	2	0
70	56	1 s	4	0	1 s	4	0	1 s	4	0
270	76	12 s	2	0	12 s	2	0	6 m 22 s	2	0
404	122	44 m	2	0	44 s	2	0	1 h 14 m	2	0
113.4	34.7	7 s	3.5	4.2	13 s	3.6	8.5	7 m 34 s	3.5	10

Note: *t*, *r* and *c* represent, respectively, the running time, the reticulation number and the redundant cluster number.

**Table 3:** Results of BIMLR, LNETWORK and CASS for the practical datasets.

Data		BIMLR			LNETWORK			CASS		
|C|	|X|	*t*	*r*	*c*	*t*	*r*	*c*	*t*	*r*	*c*
86	37	3 s	8	23	13 s	8	11	3 s	8	27
38	20	2 s	6	25	6 s	6	15	4 s	6	25
43	22	1 s	5	11	1 s	5	3	1 s	4	12
72	27	3 s	7	29	1 s	7	19	15 s	7	43
52	22	9 s	8	15	1 m 12 s	8	15	17 s	7	33
79	27	2 m 40 s	10	52	39 s	8	44	7 m 21 s	8	89
38	16	5 s	8	25	14 m 12 s	9	36	15 s	7	50
41	16	1 s	5	7	3 s	5	4	2 s	5	29
12	8	1 s	2	0	1 s	2	0	0 s	2	2
45	20	4 s	7	47	31 s	7	28	4 h 14 m	7	66
22	11	1 s	3	4	1 s	3	1	0 s	3	5
17	10	1 s	3	7	1 s	3	4	1 s	3	8
46	16	9 s	8	22	21 s	8	15	23 s	7	34
22	11	3 s	5	21	10 s	4	13	2 s	4	23
22	10	2 s	5	19	10 s	4	12	2 s	4	21
42.3	18.2	14 s	6	20.5	1 m 11 s	5.8	14.7	18 m	5.5	31

Note: *t*, *r* and *c* are defined as in Table 2.

The papers [31, 32] have shown that the LNETWORK and the BIMLR are superior to the CASS in terms of the influence of input data order; and the two methods are faster than the CASS. Here we just compare the LNETWORK and the BIMLR on the influence of input data order, and the results are shown in Table 1. In the experiment, because each program needs to construct the network for every input order of dataset, the running time is factorial. Accordingly, here we just use the dataset with small scale. When comparing networks constructed by a method for the same dataset with different input orders, the difference of those networks is more small, the method is more stable, otherwise it is unstable. Here the difference among the constructed networks is measured by means of the tripartition distances [34].

Table 1 shows the number of constructed networks, the mean, the minimum (min) and the maximum (max) of tripartition distance of those networks constructed for each dataset. And the last row shows the average values. From the Table 1 we come to the following conclusions. First, for the same data with different input orders, the number of different networks constructed by the BIMLR is less than the number of different networks constructed by the LNETWORK except for the dataset with |C|=21 and |X|=11. Second, for almost datasets, the mean, min, max of the BIMLR are less than corresponding values of the LNETWORK except for the datasets with |C|=22,|X|=13, |C|=21,|X|=11 and |C|=22,|X|=10. The result shows that, when the input order of the data is changed, even the network constructed by the BIMLR is more than one, those networks are more similar to each other than the networks constructed by LNETWORK. Thus, the BIMLR are more stable than the LNETWORK.

We compare the BIMLR, the LNETWORK and the CASS on several artificial datasets. Table 2 shows the results, which have the reticulation number *r*, the redundant cluster number *c*, the running time *t* in hours (h), minutes (m) and seconds (s), and their average values in last row. Table 2 shows that the BIMLR takes least time except for the datasets with |C|=30,|X|=5 and |C|=404,|X|=122. For every dataset, the reticulation number of the network constructed by the BIMLR is the same as that of the network constructed by CASS, which is less than that of the network constructed by the LNETWORK. The networks constructed by LNETWORK have fewer redundant clusters than the networks constructed by CASS for almost all datasets, while the networks constructed by BIMLR have fewest redundant clusters. Thus, the networks constructed by BIMLR are simplest in terms of the redundant clusters and the reticulation number contained in the constructed networks for the artificial datasets.

We compare the BIMLR, the LNETWORK and the CASS on practical datasets. Table 3 shows the results, which have the reticulation number *r*, the redundant cluster number *c*, the running time *t* in hours (h), minutes (m) and seconds (s) and their average values in last row. Table 3 shows the BIMLR takes least time except for the dataset with |C|=79,|X|=27. For most datasets, the reticulation number of the networks constructed by the CASS is least, secondly LNETWORK, at least BIMLR. The networks constructed by LNETWORK have fewest redundant clusters, secondly the BIMLR, at least CASS. Table 3 shows that the average reticulation number of LNETWORK and BIMLR is slightly more than that of CASS. Hence, the networks constructed by LNETWORK are simplest for the practical dataset.

From the Tables 2 and 3, it follows that the LNETWORK and the BIMLR take less time than the CASS; the networks constructed by LNETWORK and BIMLR have fewer redundant clusters than those constructed by CASS; and the average reticulation number of LNETWORK and BIMLR are slightly more than that of CASS.

## Conclusion

We compared BIMLR, LNETWORK and CASS using one artificial and one practical dataset. The results show that the BIMLR is superior to the others for the artificial datasets, while the LNETWORK is superior to the others for the practical datsets. Accordingly in practice, the LNETWORK is the best option for the construction of networks.
